# Developing a system dynamics model for prediction of phosphorus in facultative stabilization ponds

**DOI:** 10.1186/s13568-019-0882-6

**Published:** 2019-09-25

**Authors:** Ebrahim Shahsavani, Ali Asghar Ebrahimi, Mohammad Hassan Ehrampoush, Houshang Maleknia, Hadi Eslami, Mohammad Reza Samaei

**Affiliations:** 1 Research Center for Social Determinates of Health, Jahrom University of Medical Sciences, Jahrom, Iran; 2 Environmental Science and Technology Research Center, Department of Environmental Health Engineering, Shahid Sadoughi University of Medical Sciences, Yazd, Iran; 3 Department of Environmental Health Engineering, School of Health, Shiraz University of Medical Sciences, Shiraz, Iran; 4 Occupational Environment Research Center, Department of Environmental Health Engineering, School of Health, Rafsanjan University of Medical Sceiences, Rafsanjan, Iran

**Keywords:** Phosphorus modeling, System dynamics modeling, Wastewater stabilization ponds, Facultative ponds

## Abstract

System dynamics is considered as a computer-aided approach to policy analysis and design. It includes the response and reaction of a system to external shocks. In the present research, following the sampling and testing phases, a system dynamics model was developed for modeling of phosphorus in facultative stabilization ponds. First, the scheme of soluble reactive phosphorous stock, its specifications and parameters were determined and created in the VenSim PLE 7.1 software. Then, mathematical relations were determined for each process. Finally, the model was calibrated and verified based on the data from the Yazd facultative ponds, Iran. Sensitivity analysis showed that the most important factors affecting phosphorus concentration in the ponds are the phosphorus settling rate, losses caused by algal respiration and excretion, while the losses caused by herbivorous zooplanktons, hydrolysis rate of inorganic carbon, and ratio of phosphorus to chlorophyll-a had the least importance. Results showed that, algal growth rate and phosphorus settling rate were important factors in phosphorus removal. Hence, with appropriate retention time in the pond, it can be managed more properly. The ratio of phosphorus to algae had less importance in the model. The ratio of carbon to phosphorus and rate of respiration of carnivorous zooplanktons did not affect the phosphorus concentration. It is recommended that this model can be used for pond management and overall assessment of facultative ponds.

## Introduction

Wastewater stabilization ponds (WSPs) are the simplest and most widespread wastewater treatment plants for stabilizing biodegradable materials (Beran and Kargi 2005; Sah et al. 2011). WSPs provide suitable conditions for natural wastewater treatment processes by natural forces such as sunlight, wind, temperature, wild plants, and animal life (Samaei et al. 2016a). WSPs employ the most basic methods for wastewater treatment in developing countries, especially in the hot climate region (Møller et al. 2016; Shahi et al. 2013). WSPs, commonly known as lagoons, consist of a combination of three different types of ponds such as anaerobic (AP), facultative (FP) and maturation ponds (MP) (Sah et al. 2012). These ponds have been developed with an aim to produce effluent that is suitable for discharge to the environment or reuse for different purposes with the low cost and simple operation (Abbas et al. 2006; Naddafi et al. 2009; Shilton and Mara 2005). Wastewater is treated in WSPs as a result of algae and bacteria symbiosis and others complex ecosystem such as virus, protozoa, rotifers, insects, crustaceans, and fungi (Kehl et al. 2009; Olukanni and Ducoste 2011). Organic materials in wastewater are biodegraded by aerobic bacteria and dissolved oxygen required to bacteria were provided by algal photosynthesis (Eslami et al. 2017; Martin and Vanrolleghem 2014). Facultative ponds (FPs) are very complex because of the three anaerobic, aerobic and facultative zones (Ebrahimi et al. 2010). Upper layer provides aerobic conditions owing to presence of dissolved oxygen while lower layer has anaerobic conditions as a result of lack of dissolved oxygen (Sah et al. 2011). An intermediate layer is also located between the aerobic and anaerobic layers. FPs have usually depths of 1.5–2.5 m and their retention time is 30 to 70 days (Sah et al. 2011).

Although the use of WSPs and FPs in recent years has been of great interest to researchers for the sake of simplicity and maintenance, the mechanism for the elimination of many pollutants such as phosphorus in the WSPs has not been fully elucidated. Recently, modeling has been used as a low-cost tool to describe and better understanding the mechanism of the process of removing pollutants in WSPs (Eslami et al. 2018a; Sah et al. 2012).

Today, various models are used to model the removal of phosphorus from sewage (Eslami et al. 2018b; Hauduc et al. 2015; Mbamba et al. 2016), but compelling environmental models should have special features to solve conflicts between institutions, groups and organizations that are involved in environmental quality. A good model must be powerful, adaptable and accessible (Mohammadi et al. 2019; Samaei et al. 2016b). Capabilities of such models include applicability, utilization of new sciences and competitive advantage (RadFard et al. 2019; Samaei et al. 2010). Adaptability refers to the degree of compliance of the model with the outside world. Accessibility embraces the following capabilities: being user-friendly, and cost-effective (Hannon and Ruth 2014; Rahaman et al. 2014). Riversa et al. used system dynamics approach to predict the release of nutrients from the Peel–Harvey catchment in South Western Australia. They used Stella’s software as a model-making tool. They concluded that basin management, regardless of phosphorus, is not possible (Rivers et al. 2013). Chu et al. studied application of system dynamics on shallow lakes in Taiwan. They have concluded that SD can help us to predict and understand temporally in sequential planning for water supply and environmental protection (Chu et al. 2010). Numerous studies have focused on the modeling of WSPs (Benedetti et al. 2010; Craggs et al. 2004; Rivas et al. 2008; Senzia et al. 2002). Moreover, limit studies have also addressed modeling based on the system dynamics method and nutrients (Martin and Vanrolleghem 2014; Mesplé et al. 1996). In a study conducted in vitro, the results showed that Dynamics Models can be used to determine the purification efficiency of stabilized ponds. In the system dynamics model (SDM), the following four components are used to develop a model and demonstrate casual relationships: Stocks or state variables, Flows, Arrows or links and Convertors or parameters (Samaei et al. 2010). State variable shows the state of the ecosystem of concern. The objectives of SDM are understanding the mechanisms and the reasons for dynamics of systems, as well as, finding appropriate management policies in order to improve the environmental conditions (Sun et al. 2017).

Compared to other approaches, SDM needs less data for facultative ponds, it is cost-effective and can yield satisfactory results (Nazareth and Choi 2015). Therefore, the objective of this study is to create and develop an appropriate model for phosphorus modeling using the system dynamics approach. In this study, the VenSim, which is a system dynamics tools, is employed and a system dynamics model developed for phosphorous modeling at facultative ponds.

## Materials and methods

### System overview

**Fig. 1:**
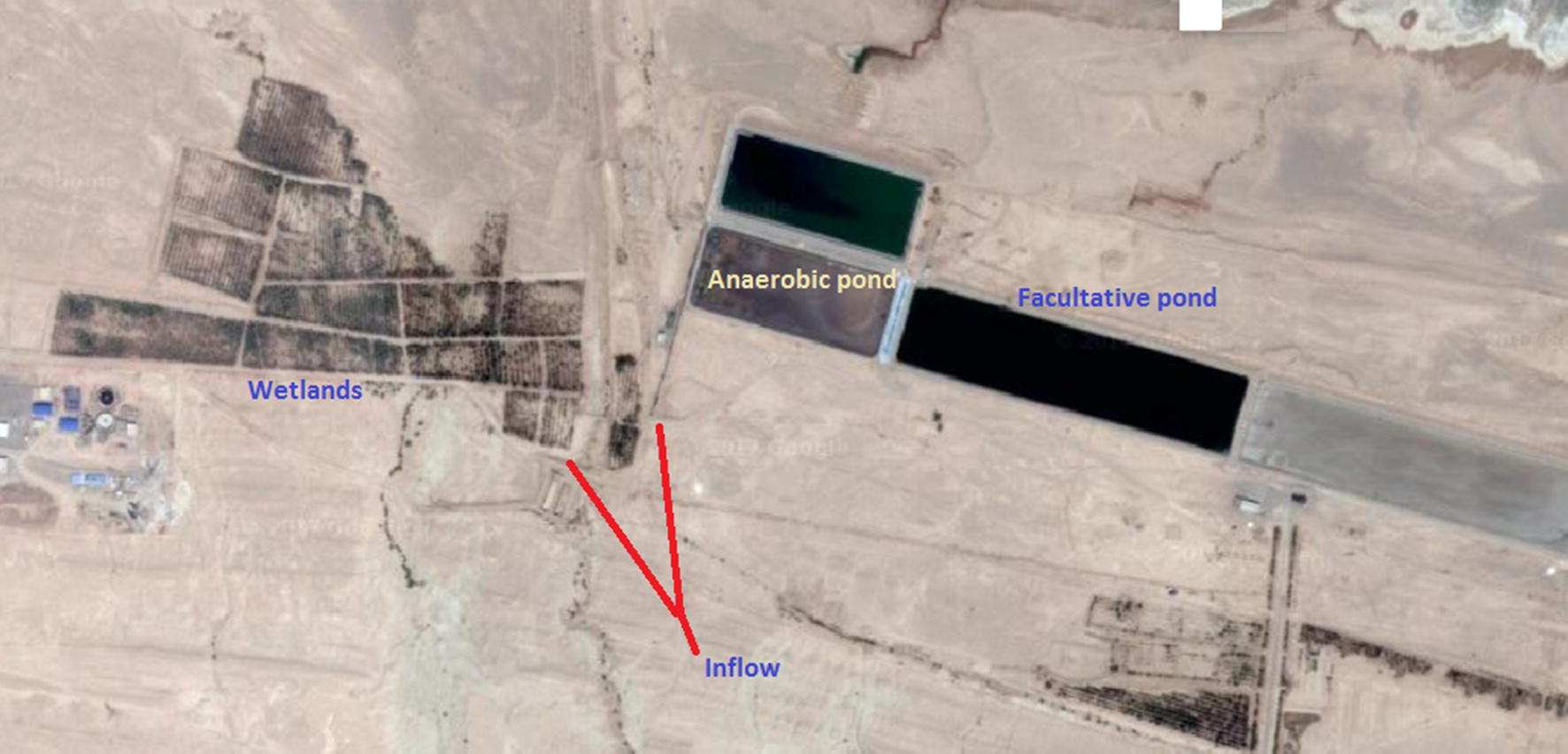
The position of wastewater stabilized ponds (WSPs) and artificial wetlands of Yazd wastewater treatment plant

As seen in Fig. 1, the Yazd’s wastewater is divided into two parts after entering the treatment plant: One section enters the anaerobic pond and the other part enters a Wetland (fixed: 15 L/s). A Yazd wastewater stabilization pond includes the following units: screening, anaerobic pond and two secondary facultative ponds in series. Hydraulic time in aerobic ponds is 5 days. Each pond has 400 m length, 100 m width and 5 m depth. However, since the first facultative pond has the potential for wastewater treatment, sampling and modeling was done in this pond.

In the first path after entering the anaerobic pond and passing through it, the sewage stream enters to the facultative pond.

### Sampling and analysis methods

In this study, sampling was performed instantaneously from WSPs in Yazd wastewater treatment plant. Sampling was carried out weekly for 92 day (from April 21st to July 22nd, 2010). Samples were taken from a few points (three points at the facultative pond entrance, three points at the facultative pond outlet, nine points in the surface layer (depth of 15 cm) of the pond, and nine points in the deep layer (depth of 1 m) of the pond). Samples were analyzed immediately in the laboratory. A boat was used for taking the samples from the facultative pond. Special containers were used to take samples. Therefore, a total of 18 samples were taken from 9 surface points and 9 points at depth. Samples of each section were mixed together and composite samples were created. Then, a composite sample was taken from the surface, a composite sample was taken from the outlet and a composite sample was taken from the surface and deep layers in the facultative pond. It was tried to fix sampling points to study the trend of parameters changes. Samples were analyzed in the laboratory. Then the values for the lower and upper layers were averaged. Concentration of soluble reactive phosphorous (SRP) was measured at different times with the standard method (once a week in 92 day). At the analysis stage, some of the parameters such as pH, DO, EC, and temperature measured immediately at the site. The other parameters such as NH_4_-N, NO_3_-N, COD, SCOD and SRP measured according to standard methods in the laboratory (Rice et al. 2012). Here, these methods are briefly described. pH, EC, and temperature were measured using the HATCH device (Model: ION 156), DO was measured by DO meter (Model: CEYER SCAN). The nitrate (NO_3_-N as mg/L) was measured by colorimetric method (using NITRATE powder packets) and with the DR 2000 (HATCH, Germany). SRP (PO_4_ as mg/L) was measured using a colorimetric method of ammonium molybdate and tin chlorine and measured with the DR 2000 apparatus. Ammonia (NH_3_ as mg/L) was measured using zinc sulfate colorimetric method and a Nessler reagent with the DR 2000 apparatus. COD and SCOD measured as mg/L with closed reflux method (Method: 5220C and D). To measure SCOD, samples were passed through a 45 μm filter (Rice et al. 2012).

### Model design

The model presented in this research discusses a simulation tool which estimates SRP in Yazd wastewater stabilization ponds based on the system dynamics approach. In order to simplify the model, the pond is considered as a continuous stirred rank reactor (CSTR) (Kehl et al. 2009; Samaei et al. 2016b). The structure of the actual model in detail as programmed into VenSim^®^ PLE 7.1. After discovering the relationship between SRP and each state variable, all of the parameters and influential variables were identified and illustrated schematically in the VenSim software. Next, the directions of the relations were determined. In other words, the SRP variable was defined in the form of the four major components of system dynamics and was shown in the VenSim design screen.

Afterwards, mathematical relations were identified for each link. The first mass equation was also written for each state variable. In general, the concentration of each chemical in the facultative pond can be expressed as Eq. (1):

1 $${\text{Concentration}} \, \left( {\text{mass}} \right) = {{\text{input}}{-}{\text{output}}} + {\text{Resuspension}}-{\text{settling}} \pm {\text{reaction}} .$$

Input stands for the level of the pollutant of concern (SRP) in the inflow of wastewater transferred from the anaerobic pond to the facultative pond. The output shows the level of pollutant in the outflow from the facultative pond. Considering the principle of mass balance, Eq. 1 can be written as Eq. 2 (Samaei et al. 2016b):

2 $$V\frac{{dp_{s} }}{dt} = a_{pc} k_{h} (T)VC_{d} + a_{pa} k_{ra} (T)Va + a_{pc} k_{rh} (T)V_{zh} + a_{pc} k_{rc} (T)VZ_{c} - a_{pa} k(T,n_{t} ,p_{s} ,I)Va.$$

The characteristic of the parameters in Eq. 2 was described in Table 2. The effect of temperature on chemical reactions can be investigated using various approaches. In the present study, the van’t Hoff-Arrhenius equation has been used (Samaei et al. 2016b). Due to the large area of the pond, the study also considers the evaporation and precipitation factors, as they can affect the volume of the pond and other wastewater quality parameters. Precipitation and evaporation information were taken from the Yazd meteorological office. In the next phase, the inflow and outflow and light intensity were put into the model as lookup. By these tables, the relationship between the inflows of wastewater from the anaerobic pond to the facultative pond provided to the model. Also some relevant time series were developed. In other words, the values of some variables were introduced into the model in a time-dependent manner, rather than in a constant manner. The process was also accomplished for some other variables such as light intensity and measured amounts of variables. The model for this research considers the facultative pond as a continuous stirred-tank reactor (CSTR). There are three important steps to the modeling process that include verification, calibration and validation (Kohn et al. 2016). After the creation of their variables and their relationships on the Vensim page, the corresponding equations were written. Then validation of the model was done. The model was verified using the measured data of the facultative pond. Afterwards, the Yazd facultative pond was calibrated in 83 days. Then, the sensitivity analysis of the model was performed. For this purpose, some kinds of parameters and\or kinetic coefficients were changed and their effect on the state variable was investigated. Table 1 presents parameters of the estimated model, which include variable name, symbols and units.

**Table 1:** Parameters, symbols and units used in the model

Variable name	Symbol	Unit
Algae	A	mgChl-a/m^3^
Ratio of phosphorus to carbon	A_pc_	mgPgC^−1^
Ratio of phosphorus to chlorophyll	Apa	mgPchla
Solar radiation	I	Lyd^−1^
Volume	V	m^3^
Combined decline of respiration and excretion	k_ra_	d^−1^
Half-saturation constant for precedence of ammonium	k_am_	d^−1^
Decline rate	Kd^−1^	d^−1^
Ammoniums’ nitrogen	n_a_	mgNm^−3^
Nitrate nitrogen	n_i_	mgNm^−3^
Soluble reactive phosphorus	SRP	mgPm^−3^
Time	Time	d
Carbon hydrolysis rate	Kh	d^−1^
Decline caused by herbivorous zooplanktons	Krh	d^−1^
Carnivorous zooplanktons	Zc	mgCm^−3^
Herbivorous zooplankton	Zh	mgCm^−3^
Soluble reactive phosphorus	Ps	mgPm^−3^
Soluble inorganic carbon	Cd	gCm^−3^

## Results

**Fig. 2:**
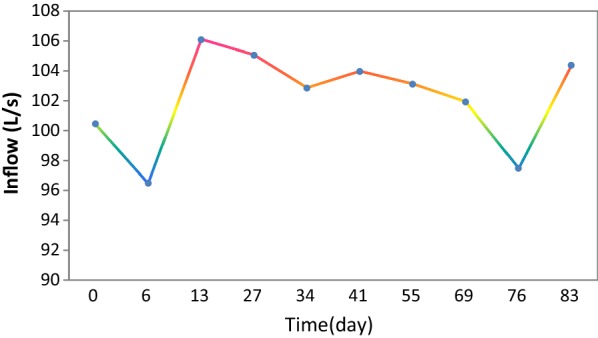
Inflow of wastewater treatment plant during various days in the model

The wastewater inflow in different days of modeling duration time (92 day) was shown in Fig. 2. These values have been taken from the plant’s authorities.

According to the calculations, the amount of phosphorus loaded to the treatment plant was about 430 kg per day (156 ton/year). The rainfall and evaporation in Yazd are 60 and 3200 mm per year, respectively (Ebrahimi et al. 2010). These amounts of precipitation and evaporation are variable throughout the year and were considered as a lookup in the model.

**Fig. 3:**
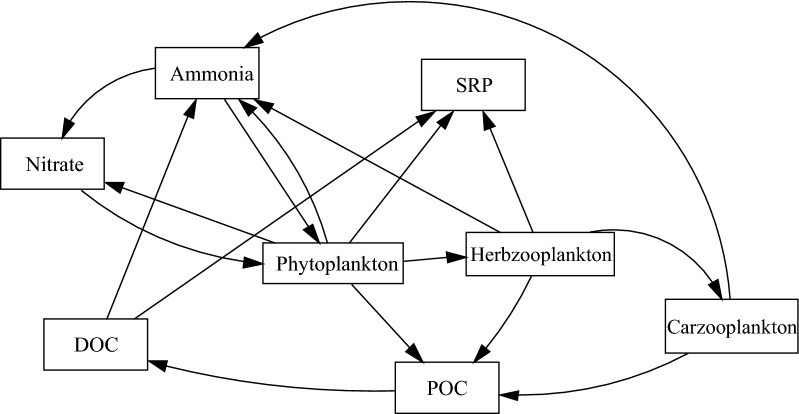
General relationships diagram between stocks in the model

**Fig. 4:**
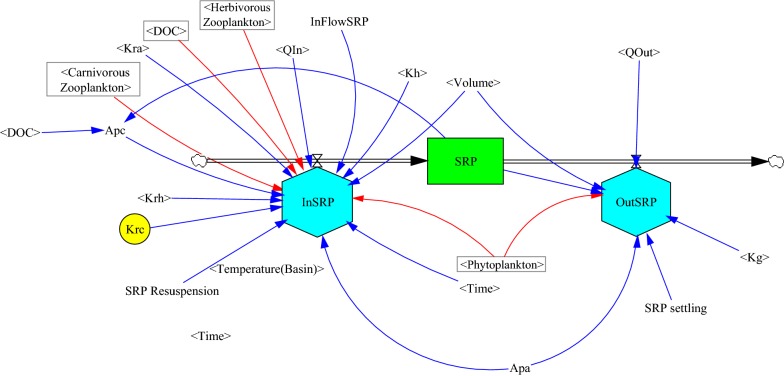
Schematic diagram of soluble reactive phosphorus dynamics model

In this model, various stocks are considered. General relationships diagram between stocks in the model was shown in Fig. 3. Figure 4 shows the schematic diagram of the SDM for SRP. In Fig. 4, the schematic diagram of SRP stock and the factors affecting it are shown. As seen in this figure, factors such as resuspension of phosphorus and levels of input phosphorus lead to an increase in the levels of soluble reactive phosphorus while factors such as the level of output phosphorus and settling of phosphorus lead to a reduction in the content of the SRP as a result of suspended algae and settling of algae.

**Fig. 5:**
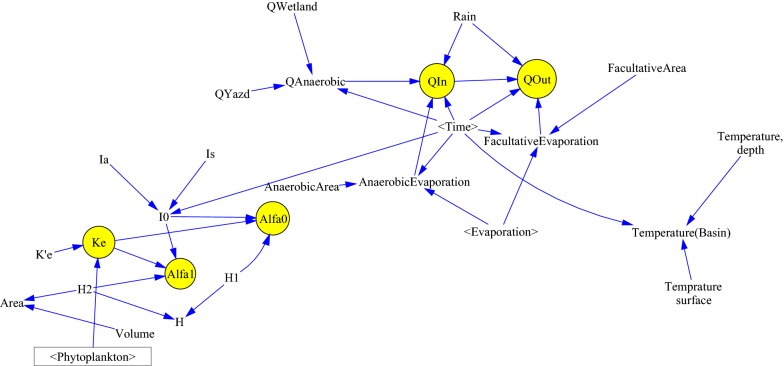
Schematic diagram of the relationships between inflow, temperature, light, and evaporation in the model

Figure 5 shows the graphical diagram of the causal relationships between flows, temperature, light coefficients, and evaporation together and with the physical characteristics of the pond, such as area and depth in the model.

**Fig. 6:**
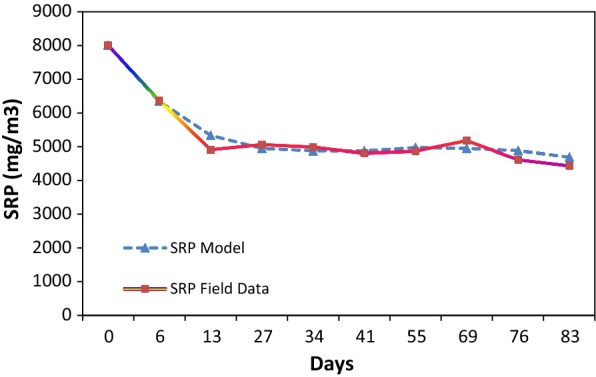
Comparison of modeled results of soluble reactive phosphorus and measured data (mg/m^3^) during 83 days

Figure 6 shows the comparison between the measured values of SRP and model estimated values. After calibration phase, coefficients, rates, and parameters for facultative pond obtained. Summary of major model components were described in Table 2. In order to done a sensitivity analysis, it is necessary to reduce or increase one of the coefficients in order to study its effect on model estimation.

**Table 2:** Calibration coefficients, rates and parameters in the facultative pond model

Parameter	Symbol	Value	Unit
Volume	v	80,000	m^3^
Single degree growth rate of algae	kg	0.35	d^−1^
Hydrolysis rate	Kh	–	d^−1^
Hydrolysis rate in 20°	Kh, 20	0.97	d^−1^
Kh temperature correction factor	Kh, Teta	1.06	–
Decline caused by respiration	K_rc_	0.072	d^−1^
Decline caused by herbivorous zooplanktons in 20°	K_rh, 20_	1.8	d^−1^
K_rh_ temperature correction factor	K_rh, Teta_	1.08	–
Ratio of phosphorus to chlorophyll	A_pa_	0.15	mgPmgChLa^−1^
Ratio of phosphorus to carbon	A_pc_	0.8–0.22	–
Decline caused by the combined effect of respiration and excretion	K_ra_	0.05–0.9	d^−1^

## Discussion

There are many challenges and conflicts on how to improve the environment between environmental activists with government officials. This is more evident among developing countries. So using models can help these groups make better decisions (Martin and Vanrolleghem 2014). Such decision models must have certain characteristics. They should, as a matter of simplicity, provide good information to decision makers. A good model should be powerful, adaptable, accessible, user friendly, cost effective and flexible (Samaei et al. 2016b). In this research a mathematical model was developed to simulate the phosphorus in WSPs. The model was based on the system dynamics approach. For sensitivity analysis of the model, in the VenSim software some parameters and\or kinetic coefficients were changed and their effect on the state variable was investigated. Findings of the sensitivity analysis of the Yazd WSP model show that k_g_ (algae growth rate), the phosphorus settling rate (SRP settling) and K_ra_ (declination caused by the combined effect of algal respiration and excretion) were the most important in the model. Proper control of phosphorus levels is required to control algal growth (Beran and Kargi 2005). Moreover, more algal growth, more pond efficiency (Schmidt et al. 2017). Powell et al. studied on Luxury uptake of phosphorus by microalgae in WSP and the results show that the phosphorus was consumed during algal growth and resulting phosphorus removal in WSP (Powell et al. 2011). Therefore, in order to improve the efficiency of facultative ponds and manage the amount of algae produced, the level of phosphorus in the pond is most important. Because settling of phosphorus and resuspension of phosphorus from sediments are very important in managing of phosphorus, it is recommended to pay special attention to this when designing the dimensions of the pond, especially the depth selection. The results of study conducted by Schmidt and et al., show that the best depth for algal growth and phosphorus removal in WSP was less than 2 m (Schmidt et al. 2017). It is also suggested that the sediments be discharged from a pond with a regular schedule.

K_rh_ (decline caused by herbivorous zooplanktons) and A_pa_ (ratio of phosphorus to chlorophyll-a) had the least importance in the model. The sensitivity analysis revealed that the decline caused by herbivorous zooplanktons and the ratio of phosphorus to chlorophyll-a were the least important factor in the model. It is worth mentioning that chlorophyll-a indicates photosynthesis of algae and the model is less influenced by fluctuations of input phosphorus. Since the amount of phosphorus in the pond was sufficient enough, it is not a limiting factor for algae growth. But the amount of phosphorus can increase algae growth.

K_rc_ (ratio of respiration of carnivorous zooplanktons), A_pc_ (ratio of phosphorus to carbon), K_am_ (half-saturation constant for preferential of ammonium), and F_am_ (a part of inorganic nitrogen separated from the pond through algal uptake) are among the insignificant factors in the model. Therefore, shortage of carnivorous zooplanktons (that feed on herbivorous zooplanktons) as a result of their respiration is not of great importance to the model and the system is not limited in this regard. Variations of the ratio of phosphorus to carbon do not influence model equilibrium either. The half-saturation constant for preferential of ammonium and a part of inorganic nitrogen, which is separated from the pond through algal uptake, are among the insignificant factors in the phosphorus model. Therefore, fluctuations of these parameters do not affect values of phosphorus estimated by the model. Moreover, temperature of pond is an important factor. On the other hand, light intensity is an insignificant factor. Study by Schmidt et al., indicated that temperature and photosynthetically active radiation were positive and negative effects on phosphorus uptake in WSP (Schmidt et al. 2016). Yazd is located 32° latitude in the warm and dry region of Iran. The distance to the Persian Gulf is 475 km. In this latitude, the intensity of light is high. Also, in most days of the year, the weather is sunny (Ebrahimi et al. 2010). This is probably why the intensity of the light in the model was not significantly affected. The temperature has a lot of effect on the model. Therefore, the pond must have optimal temperature in order to the system be able to function properly.

Therefore, system dynamics models are provided for overall assessment of the quality of facultative ponds. The paper has presented a new modeling approach for the study of phosphorus in waste stabilization ponds. The model that was developed in this study is very simple and flexible, so it’s easy to add new parameters or remove some of it. In general, the use of this model is proposed to predict the status of facultative ponds. The results of modeling with this method showed that the use of system dynamics models can provide overall and adequate information to authorities so that they can decide well for choosing different environmental improvement options. In this model, the phosphorus settling rate is an important factor in reducing phosphorus. Hence, satisfactory retention time in the facultative pond contributes to better management of the pond. The ecological system dynamics model is useful for pond management; however, it is better to use two and three dimensional models for a more accurate evaluation.

## Data Availability

The data supporting our finding included in the manuscript.
